# Local transplantation of mesenchymal stem cells improves encephalo-myo-synangiosis-mediated collateral neovascularization in chronic brain ischemia

**DOI:** 10.1186/s13287-023-03465-7

**Published:** 2023-09-04

**Authors:** Xincheng Zhang, Yimin Huang, Yuan Liu, Yanchao Liu, Xuejun He, Xiaopeng Ma, Chao Gan, Xin Zou, Sheng Wang, Kai Shu, Ting Lei, Huaqiu Zhang

**Affiliations:** 1 Department of Neurosurgery, Tongji Hospital of Tongji Medical College of Huazhong University of Science and Technology, Jiefang Avenue 1095, Wuhan, 430030 Hubei Province China; 2 Institute of Integrated Traditional Chinese and Western Medicine, Tongji Hospital of Tongji Medical College of Huazhong University of Science and Technology, Wuhan, 430030 China

**Keywords:** Mesenchymal stem cells, Encephalo-myo-synangiosis, Angiogenesis, Moyamoya disease, Cell transplantation

## Abstract

**Background:**

To explore whether local transplantation of mesenchymal stem cells (MSCs) in temporal muscle can promote collateral angiogenesis and to analyze its main mechanisms of promoting angiogenesis.

**Methods:**

Bilateral carotid artery stenosis (BCAS) treated mice were administrated with encephalo-myo-synangiosis (EMS), and bone marrow mesenchymal stem cells (BMSCs) were transplanted into the temporal muscle near the cerebral cortex. On the 30th day after EMS, the Morris water maze, immunofluorescence, laser speckle imaging, and light sheet microscopy were performed to evaluate angiogenesis; In addition, rats with bilateral common carotid artery occlusion were also followed by EMS surgery, and BMSCs from GFP reporter rats were transplanted into the temporal muscle to observe the survival time of BMSCs. Then, the concentrated BMSC-derived conditioned medium (BMSC-CM) was used to stimulate HUVECs and BMECs for ki-67 immunocytochemistry, CCK-8, transwell and chick chorioallantoic membrane assays. Finally, the cortical tissue near the temporal muscle was extracted after EMS, and proteome profiler (angiogenesis array) as well as RT-qPCR of mRNA or miRNA was performed.

**Results:**

The results of the Morris water maze 30 days after BMSC transplantation in BCAS mice during the EMS operation, showed that the cognitive impairment in the BCAS + EMS + BMSC group was alleviated (*P* < 0.05). The results of immunofluorescence, laser speckle imaging, and light sheet microscopy showed that the number of blood vessels, blood flow and astrocytes increased in the BCAS + EMS + BMSC group (*P* < 0.05). The BMSCs of GFP reporter rats were applied to EMS and showed that the transplanted BMSCs could survive for up to 14 days. Then, the results of ki-67 immunocytochemistry, CCK-8 and transwell assays showed that the concentrated BMSC-CM could promote the proliferation and migration of HUVECs and BMECs (*P* < 0.05). Finally, the results of proteome profiler (angiogenesis array) in the cerebral cortex showed that the several pro-angiogenesis factors (such as MMP-3, MMP-9, IGFBP-2 or IGFBP-3) were notably highly expressed in MSC transplantation group compared to others.

**Conclusions:**

Local MSCs transplantation together with EMS surgery can promote angiogenesis and cognitive behavior in chronic brain ischemia mice. Our study illustrated that MSC local transplantation can be the potential therapeutical option for improving EMS treatment efficiency which might be translated into clinical application.

**Supplementary Information:**

The online version contains supplementary material available at 10.1186/s13287-023-03465-7.

## Background

Moyamoya disease is a cerebrovascular disease with an unclear etiology that mainly occurs in East Asia. Its characteristic lesion is progressive stenosis at the end of the bilateral internal carotid arteries, the anterior cerebral arteries, and the beginning of the middle cerebral arteries, accompanied by abnormal vascular network formation at the skull base [1, 2]. There is still a lack of effective drug treatments for moyamoya disease, and vascular reconstruction surgery is the main treatment method. The main methods of surgery include direct bypass surgery and indirect bypass surgery [3]. Currently, most centers perform both direct and indirect bypass surgery in one surgery and combine the advantages of both, known as combined vascular reconstruction surgery [4, 5].

Encephalo-myo-synangiosis (EMS) is a widely used indirect bypass surgery for Moyamoya disease and is also a part of combined vascular reconstruction surgery [6–8]. The temporal muscle of the patient was dissected and covered on the cerebral hemispheric surface with insufficient perfusion in this surgery. The principle of the surgery is to induce neovascularization of the temporal muscle and supply blood to the brain to alleviate the degree of cerebral ischemia [9]. However, some of the cohort studies showed that a certain proportion of the patients who underwent EMS surgery failed to achieve the expected results. Failure to achieve the expected results of EMS surgery is potentially due to effective collateral circulation between the temporal muscle and the cerebral hemisphere not being well established [10, 11]. Therefore, promoting the collateral circulation of EMS will largely improve the outcome for these patients.

Mesenchymal stem cells (MSCs) are multipotent cells that can differentiate into various cell types, including endothelial cells, chondrocytes, myocytes, adipocytes, and even glial cells [12, 13]. Furthermore, an increasing number of studies have suggested that MSCs exhibit an active treatment effect against various disorders, including inflammation, neuron degeneration diseases, cancers, ischemia diseases, etc. [14–17]. Studying the underlying mechanisms of this omnipotent therapeutic effect has become a hot topic. Some research has shown that MSCs may release many cytokine factors and noncoding RNAs, such as miRNAs and lncRNAs, which can effectively promote angiogenesis [18, 19]. We speculate that MSCs may also be helpful in improving collateral neovascularization after EMS.

In the present study, we transplanted MSCs onto the temporal muscle during EMS surgery and evaluated the promoting effect on local angiogenesis and the improvement of cognitive behavior with chronic brain ischemia in the BCAS mouse model.

## Materials and methods

### Animals

All animal procedures performed in this study were specifically approved by The Experimental Animal Ethics Committee of Huazhong University of Science and Technology and the animal experiments were complied with the "Animal Research: Reporting In Vivo Experiments" (ARRIVE) guidelines. Male C57BL/10ScNJ mice (age, 2–3 weeks, 8–10 weeks; weight, 4–8 g, 21–25 g) and male Sprague‒Dawley (SD) rats (250–280 g) were obtained from GemPharmatech Co., Ltd. SD-Tg (GFP) rats (100–150 g) were given as a gift by Dr. Wang Yu. The mice were fed in The Experimental Animal Center of Tongji Scientific Building, Tongji Hospital. All mice were maintained in a controlled environment at 22 ± 3 °C and 60% relative humidity under a 12-h light/dark cycle and were given free access to standard rodent nutrition and water.

### The harvest and culture of BMSCs

The bone slice method was used for the extraction of primary MSCs from mice [20], and the whole bone marrow culture method was used for the extraction of primary MSCs from rats.

For mice, 2-week-old mice were sacrificed by cervical dislocation, the femur was isolated under sterile conditions, the epiphysis of the femur was cut off, and a 0.45-mm syringe needle was inserted into the bone cavity. The bone marrow was continuously flushed with α-MEM medium until the bone cavity became white. The femur was cut into small bone chips of approximately 1–3 mm^3^ and then suspended in 3 ml of α-MEM containing 10% (vol/vol) FBS in the presence of 1 mg ml^−1^ (wt/vol) collagenase II. Digest the chips for 1–2 h in a shaking incubator at 37 °C with a shaking speed of 200 rpm. After digestion, the bone chips were washed with α-MEM and seeded into a 25-cm^2^ plastic culture flask in the presence of 6 ml of α-MEM supplemented with 10% (vol/vol) FBS for culture. The medium was changed on the third day of culture, and the cells were digested and passaged on the fifth day. Cells at passages 3–8 were used for in vivo and in vitro experiments.

After the rats were sacrificed by cervical dislocation, the femur and tibia of the rats were aseptically separated, and then the epiphysis was cut off. A 21-gauge needle was inserted into the bone cavity, and the bone marrow was flushed out using a syringe filled with 5 mL DMEM/F12. The erythrocytes in the bone marrow were lysed using erythrocyte lysis buffer, and the remaining cells were collected by centrifugation at 300×*g* for 5 min and seeded in T75 culture flasks. The medium was changed on the second day of culture, and the adherent cells were digested and passaged on the fourth day.

Concentrated BMSC-CM (BMSC-derived conditioned medium): when the cell density reached 70–80%, the medium was replaced with serum-free α-MEM medium and incubated at 37 °C in a 5% CO_2_ incubator for 24 h. After 24 h, the culture medium was collected, and the mixed cells were removed by syringe filter. The filtrate was added to an ultrafiltration centrifuge tube, and the concentrated BMSC-CM was obtained by centrifugation at 8000×*g* for 30 min. As a control, the same amount of α-MEM medium was also added to an ultrafiltration centrifuge tube and centrifuged at 8000×*g* for 30 min to obtain a concentrated α-MEM medium.

### Animal models

We modified the bilateral common carotid artery stenosis (BCAS) model in mice and the bilateral common carotid artery occlusion (BCCAO) model in rats for our chronic cerebral hypoperfusion (CCH) animal model [21, 22].

The experimental animals were fasted with no food or water 8 h before the operation, and the experimental animals were placed in a room with a constant temperature. Animals were anesthetized with 5% isoflurane in oxygen with a delivery rate of 0.5 l/min for induction and 2% isoflurane for maintenance. The operation was performed after the experimental animals were completely anesthetized. The BCAS mouse model was established by exposing the left and right common carotid arteries (CCAs) one by one, detaching them from the sheath, and winding a microcoil (Piano wire diameter 0.08 mm, internal diameter 0.18 mm, coiling pitch 0.5 mm, and total length 2.5 mm; Sawane Spring Co Ltd, Japan) around each CCA. The BCCAO model in rats was achieved by carefully separating the CCA from the sympathetic and vagus nerves through an abdominal-cervical incision and double ligating both CCAs with 3–0 silk. During the whole operation, the temperature of the rats was maintained at approximately 37 °C by using a heating pad. After the procedure, mice were given buprenorphine (0.05 mg/kg) intraperitoneally to manage pain and monitored for adverse side effects.

Inclusion criteria: 1. Mice with cerebral blood flow decreased by ≥ 40% immediately after the operation as assessed by a laser speckle blood flow imaging system were included. 2. Rats with an mNSS score between ≥ 1 and ≤ 13 on the first postoperative day were included.

Exclusion criteria: 1. Mice with cerebral blood flow decreased by < 40% immediately after the operation were excluded. 2. Rats with mNSS scores < 1 and > 13 on the first postoperative day were excluded.

### EMS operation and cell transplantation

Seven days after induction of CCH, animals were placed in a stereotactic apparatus following the induction of anesthesia with isoflurane. An incision was made along the top midline of the skull, the right temporalis muscle was separated from the temporal bone through a linear incision, and after a craniectomy in the temporoparietal region, the dura was carefully opened and removed, avoiding disruption of the brain surface [23]. For implantation, cultured mesenchymal stem cells were trypsinized and resuspended in the culture medium, 5 × 10^5^ cells were suspended in 5 µl culture medium for transplantation of one mouse, and 1 × 10^6^ cells were suspended in 20 µl culture medium for the transplantation of one rat. A microsyringe (Hamilton Co., Reno, NV, USA) was used to select the angle points of a 1.5 mm square on the temporal muscle near the cortex of the brains for injection. To fix the temporalis muscle to the cerebral cortical surface after mesenchymal stem cell implantation, the overlying temporalis fascia was sutured to the contralateral aponeurosis with 6–0 silk.

A total of 273 mice were divided into the following five groups: (1) control group (n = 59); (2) BCAS group (n = 59); (3) BCAS + EMS group (n = 59); (4) BCAS + EMS + BMSC group (n = 59); and (5) BCAS + EMS + concentrated BMSC-CM group (n = 37). The mice in the control group received only neck sham surgery (only the median neck incision was performed, and the bilateral CCAs were isolated) and head sham surgery (only the median incision at the top of the skull was performed, and the temporal muscle was separated); the BCAS group mice received BCAS surgery and head sham surgery; the mice in the EMS group received neck sham surgery and EMS surgery; the mice in the BCAS + EMS + BMSC group received BCAS, EMS surgery and BMSC transplantation; and the mice in the BCAS + EMS + concentrated BMSC-CM group received BCAS, EMS surgery and concentrated BMSC-CM transplantation. Follow-up experiments were performed 30 days after EMS surgery in all experimental animals.

### Tissue preparation for immunofluorescence

Mice were anesthetized with 5% isoflurane in oxygen with a delivery rate of 0.5 l/min for induction and 2% isoflurane for maintenance. All mice were then immediately injected with an overdose of ketamine (180 mg/kg) + xylazine (30 mg/kg) for euthanasia 30 days post-EMS. The mice were transcardially perfused with PBS and 4% paraformaldehyde (PFA). Brain tissues were then isolated and placed in 4% PFA for 1 day at room temperature. The tissues were fixed with 30% sucrose for 48 h at room temperature. Frozen brain Sects. (40 µm) were sliced using a sliding microtome; the freezing platform and freezing chamber were set at -35 and -20 °C, respectively.

### Immunofluorescence

Mice were divided into four groups: (1) sham group; (2) BCAS group; (3) BCAS + EMS group; (4) BCAS + EMS + BMSC group; and (5) BCAS + EMS + concentrated BMSC-CM. All mice were injected with an overdose of ketamine (180 mg/kg) + xylazine (30 mg/kg) immediately for euthanasia 30 days post-EMS. The frozen brain sections were blocked in 5% BSA (cat. no. G5001-100G; Wuhan Servicebio Technology Co., Ltd.) with 0.1% Triton X-100 in TBS for 2 h at room temperature. Subsequently, tissues were incubated with the following primary antibodies diluted in 5% BSA in TBS overnight at 4 °C: mouse anti-glial fibrillary acidic protein (GFAP, 1:50; cat. no. ab4648; Abcam), rabbit anti-CD31 (1:5000; cat. no. ab182981; Abcam), mouse anti-CD44 (1:200; cat. no. 60224–1-Ig; Proteintech Co., Ltd.), rabbit anti-CD29 (1:200; cat. no. 12594–1-AP; Proteintech Co., Ltd.), rabbit anti-CD34 (1:200; cat. no. A7429; ABclonal Biotech Co., Ltd.), mouse anti-CD45 (1:200; cat. no. 60287-1-Ig; Proteintech Co., Ltd.) and rabbit anti-Ki67 (1:200; cat. no. ab15580; Abcam). The sections were subsequently washed three times for 10 min each in TBS and then incubated for 2 h at room temperature with the following secondary antibodies: Cy3-conjugated goat anti-mouse (1:500; cat. no. GB21301; Wuhan Servicebio Technology Co., Ltd.), Cy3-conjugated goat anti-rabbit (1:500; cat. no. GB21303; Wuhan Servicebio Technology Co., Ltd.), Alexa Fluor^®^ 488-conjugated goat anti-mouse (1:200; cat. no. GB25301; Wuhan Servicebio Technology Co., Ltd.) and Alexa Fluor^®^ 488-conjugated goat anti-rabbit (1:200; cat. no. GB25303; Wuhan Servicebio Technology Co., Ltd.) diluted in 5% BSA and DAPI (50%; cat. no. G1012; Wuhan Servicebio Technology Co., Ltd.) in TBS. The sections were washed four times (10 min/wash) with TBS before being sealed with fluorescent antifade solution (cat. no. G1401; Wuhan Servicebio Technology Co., Ltd.) and a cover glass. Images were captured using a fluorescence microscope (cat. no. CKX53; Olympus Corporation). ImageJ 1.8.0 (National Institutes of Health) software was used for data analysis.

For cells, 5 × 10^4^/ml cells were first digested and seeded into a cell culture cluster with round coverslips. After BMECs adhered, they were fixed with 4% PFA for 15 min for identification. HUVECs and BMECs were cultured in concentrated α-MEM medium and concentrated BMSC-CM for 24 h according to the above three groups and then fixed with 4% PFA, and the subsequent staining steps were the same as above.

### Morris water maze (MWM)

Mice were divided into five groups: (1) sham group; (2) BCAS group; (3) BCAS + EMS group; (4) BCAS + EMS + BMSC group; and (4) BCAS + EMS + concentrated BMSC-CM. The spatial learning and memory of the mice were tested using the MWM starting on Day 30 after EMS. The apparatus comprised a swimming pool (diameter, 1.5 m; height, 60 cm) filled with water (temperature, 21–25 °C). Mice were trained to find a submerged platform (depth, 1.5 cm) using the marker around the sidewall. During the 6-day training period, the platform was placed in the third quadrant of the testing arena. At each time trial every day, all mice were allowed 60 s to find the platform and were given a 30-s stay on the platform. At the start of each trial, the mice were randomly placed into one of the three quadrants (first, second and fourth quadrant). The average latency, moving distance, and swimming speed before locating the platform at each time trial on the test day were recorded using WMT-100 Morris software (Chengdu Taimeng Technology Co., Ltd.). On day 7, a space exploration experiment was performed, where the platform was removed and the mice were given 60 s to locate the area where the platform used to be; the duration of time spent in the target quadrant and the number of times that the mice crossed the previous platform location were recorded.

### Cell culture and concentrated BMSC-CM treatment

HUVECs and BMECs, which were obtained from the American Type Culture Collection by Tongji Hospital Nephrology Department laboratory, were presented to us in March 2022. The cells were identified by immunocytochemistry and tested for mycoplasma contamination regularly. Cells were routinely cultured in DMEM containing 10% FBS at 37 °C in an atmosphere with 5% CO2/95% air. All the experiments were performed using HUVECs and BMECs between passages 3 and 8. HUVECs and BMECs were divided into vehicle group and concentrated BMSC-CM groups. In the vehicle group, concentrated α-MEM was added to DMEM at a proportion of 5% for cell culture. In the other experimental groups, concentrated BMSC-CM was added to DMEM at different concentrations to stimulate cells.

### Cell counting kit-8 (CCK-8) assay

The viability of HUVECs and BMECs was evaluated using CCK-8 (cat. no. ST1008; Sunbao-Biotech Co., Ltd.) in accordance with the manufacturer's instructions. HUVECs and BMECs were divided into different groups, and cells were seeded into 96-well plates (cat. no. 3599; Costar; Corning, Inc.) at a density of 1 × 10^5^ cells/ml (5,000 cells/well). Different concentrations of ultrafiltrate were added to corresponding groups and incubated for 24 h. After the indicated treatments, the cells were washed with PBS, and CCK-8 reagent was added to each well at a concentration of 10 µl/ml for 4 h at 37 °C. The viability of HUVECs and BMECs was evaluated using a microplate reader (Infinite F50; Tecan Group Ltd.) at a wavelength of 450 nm.

### Transwell assay

The Transwell assay was used to analyze cell migration. HUVECs and BMECs were randomly divided into the above groups. Briefly, the upper and lower chambers were separated with polycarbonate filters (pore size, 8 µm; Corning, Inc.). DMEM without FBS and supplemented with 5% concentrated α-MEM medium, different concentrations of BMSC-CM was added to the upper chamber, and DMEM with 10% FBS was added to the lower chamber. Subsequently, HUVECs and BMECs (5 × 10^4^/ml) were seeded into the upper chamber, and both chambers were incubated at 37 °C for 48 h. Subsequently, the upper side of the membrane was fixed with 4% paraformaldehyde for 30 min at room temperature and then wiped with a cotton bud, and the cells that migrated to the lower chamber were stained with a crystal violet solution (cat. no. G1014; Wuhan Servicebio Technology Co., Ltd.) for 30 min at room temperature.

### Chick chorioallantoic membrane (CAM) assay

A CAM assay was used to assess the effect of concentrated BMSC-CM on angiogenesis [24]. Chicken eggs at seven days postfertilization, which were purchased from an ecological hatchery (Renrenjia, Shandong, China), were cleaned with 0.05% potassium permanganate, and a window of 1.0 cm^2^ was gently opened on the blunt end of the egg without damaging the embryo. A sterilized silicone ring was positioned on the CAM surface, avoiding major blood vessels. Then, 100 μL of concentrated α-MEM, 20 μL of concentrated BMSC-CM, and 100 μL of concentrated BMSC-CM were added dropwise to the silicone ring. The eggs were incubated in a forced-draft incubator at 38 ± 0.2 °C with ∼60–65% humidity. After 3 days of treatment, the vessels of the CAM were counted.

### Proteome profiler (angiogenesis array)

Angiogenesis-related proteins were assayed using the kit according to the manufacturer's instructions (Catalog: ARY015, R&D Systems, Inc.). The experiments were divided into 4 groups: (1) sham group; (2) BCAS group; (3) BCAS + EMS group; (4) BCAS + EMS + BMSC group. Brain tissue from the cerebral cortex of mice close to the side of the temporal muscle was extracted, and the tissues from the same group were mixed. 300 μg tissues were harvested from the mixed brain tissue for angiogenesis array.

### Reverse transcription-quantitative PCR (RT-qPCR)

Mice were divided into five groups: (1) control group; (2) BCAS group; (3) BCAS + EMS group; (4) BCAS + EMS + BMSC group; and (5) BCAS + EMS + concentrated BMSC-CM group. All mice were injected with an overdose of ketamine (180 mg/kg) + xylazine (30 mg/kg) immediately for euthanasia 30 days post-EMS. Total RNA was extracted from the cortex attached to the temporal muscle using TRIzol^®^ reagent (cat. no. 15596018; Invitrogen; Thermo Fisher Scientific, Co., Ltd.) according to the manufacturer's protocol, and RNA levels and quality were measured using a NanoDrop™ (Thermo Fisher Scientific, Co., Ltd.). mRNA was reverse transcribed into cDNA using Hifair^®^ III 1st Strand cDNA Synthesis Supermix (cat. no. 11141ES10; Shanghai Yeasen Biotechnology Co., Ltd.); RT was performed in a 20-µl system under the following thermocycling conditions: 25 °C for 5 min, 55 °C for 15 min and 85 °C for 5 min. qPCR was performed using Hieff^®^ qPCR SYBR^®^ Green Master mix (cat. no. 11202ES03; Shanghai Yeasen Biotechnology Co., Ltd.) and Quant Studio-1™ Design & Analysis Software (cat. no. A40426; Thermo Fisher Scientific, Inc.), under the following thermocycling conditions: one cycle at 95 °C for 5 min, followed by 40 cycles at 95 °C for 10 s and 60 °C for 30 s, and a final melt curve stage at 60 °C for 1 min and 95 °C for 1 s. The specific primers used for qPCR were as follows: Vegfa, forward, 5′-AAACGAACGTACTTGCAGATGTG-3′ and reverse, 5′-TCTTCCTTCATGTCAGGCTTTCT-3′; EGF, forward, 5′-TGATAGCCAGCTCCAATCTACTG-3′ and reverse, 5′-CCAGTCCTCTTGTTCACCCTTAT-3′; FGF1, forward, 5′-AAAGTGCGGGCGAAGTGTATATA-3′ and reverse, 5′-TTACAGCTCCCGTTCTTCTTGAG-3′; FGF2, forward, 5′-GGCTTCTGTGAGTAGTGCTTTCT-3′ and reverse, 5′-CAGTTCGTTTCAGTGCCACATAC-3′; TGF-a, forward, 5′-AAGAAGCAAGCCATCACTGC-3′ and reverse, 5′-CACTCACAGTGTTTGCGGAG-3′; TGF-b, forward, 5′-GCGGACTACTATGCTAAAGAGGT-3′ and reverse, 5′-GCTTCCCGAATGTCTGACGTATT-3′; TNF, forward, 5′-GCCTCCCTCTCATCAGTTCTATG-3′ and reverse, 5′-ACCTGGGAGTAGACAAGGTACAA-3′; IL-1a, forward, 5′-TACAGTTCTGCCATTGACCATCT-3′ and reverse, 5′-GTTGCTTGACGTTGCTGATACTG-3′. Relative target gene expression to the geometric mean of the reference gene GAPDH was determined by the Formula 2^−ΔΔCq^, where ΔCq = Cq (target gene) − Cq (GAPDH) and ΔΔCq = ΔCq (target gene) − ΔCq (control). Changes in gene expression are presented as fold changes compared to gene expression in the control group.

miRNA was reverse transcribed into cDNA using the miRNA 1st Strand cDNA Synthesis Kit (by tailing A) (cat. no. MR201; Nanjing Vazyme Biotechnology Co., Ltd.); RT was performed in a 20-µl system under the following thermocycling conditions: 37 °C for 60 min, 85 °C for 5 min. qPCR was performed using ChamQ SYBR qPCR Master Mix (High ROX Premixed) (cat. no. Q341; Nanjing Vazyme Biotechnology Co., Ltd.) and Quant Studio-1™ Design and Analysis Software (cat. no. A40426; Thermo Fisher Scientific, Inc.), under the following thermocycling conditions: one cycle at 95 °C for 30 s, followed by 40 cycles at 95 °C for 10 s and 60 °C for 30 s, and a final melt curve stage at 95 °C for 15 s, 60 °C for 60 s and 95 °C for 15 s. The specific primers used for qPCR were as follows: mmu-miR-877-5p, forward, 5′-GTAGAGGAGATGGCGCAGGG-3′; mmu-miR-21a-5p, forward, 5′-GGCTAGCTTATCAGACTGATGTTGA-3′; mmu-miR-29a-3p, forward, 5′-CTAGCACCATCTGAAATCGGTTA-3′; mmu-miR-126a-3p, forward, 5′-CTCGTACCGTGAGTAATAATGCG-3′; mmu-miR-221-3p, forward, 5′-AGCTACATTGTCTGCTGGGTTTC-3′; and mmu-U6, forward, 5′-CGCTTCGGCAGCACATATAC-3′. Relative target gene expression to the geometric mean of the reference gene mmu-U6 was determined by the Formula 2^−ΔΔCq^, where ΔCq = Cq (target gene) − Cq (mmu-U6) and ΔΔCq = ΔCq (target gene) − ΔCq (control). Changes in gene expression are presented as fold changes compared to gene expression in the control group.

### Flow cytometry

To identify whether the adherent cells were BMSCs, cells at passages 3–8 were digested with trypsin and washed with staining buffer. For 30 min, adherent cells were stained for with the following antibodies: APC/Cyanine7-CD44 (cat. no. 103027; BioLegend), FITC-CD29 (cat. no. 102205; BioLegend), PE-CD34 (cat. no. 128609; BioLegend), and APC-CD45 (cat. no. 103112; BioLegend) at 4 degrees, and unlabeled cells were used as controls. The expression of cell surface molecules was identified by flow cytometry (Cytoflex-3, Beckman Coulter).

### Statistical method

Statistical analysis of data was performed using IBM SPSS Statistics version 25.0 (IBM Software Group, Chicago, IL, USA). Data are presented as the mean ± SD, and experiments were repeated at least three times. The significant differences were determined by unpaired two-tailed Student's t test or one-way ANOVA followed by Tukey's post hoc test, and the Kruskal‒Wallis test was used to analyze nonparametric data. *P* < 0.05 was considered a statistically significant difference.

## Result

### Local application of BMSCs improves cognitive function recovery in chronic brain ischemia treated with encephalo-myo-synangiosis

**Fig. 1:**
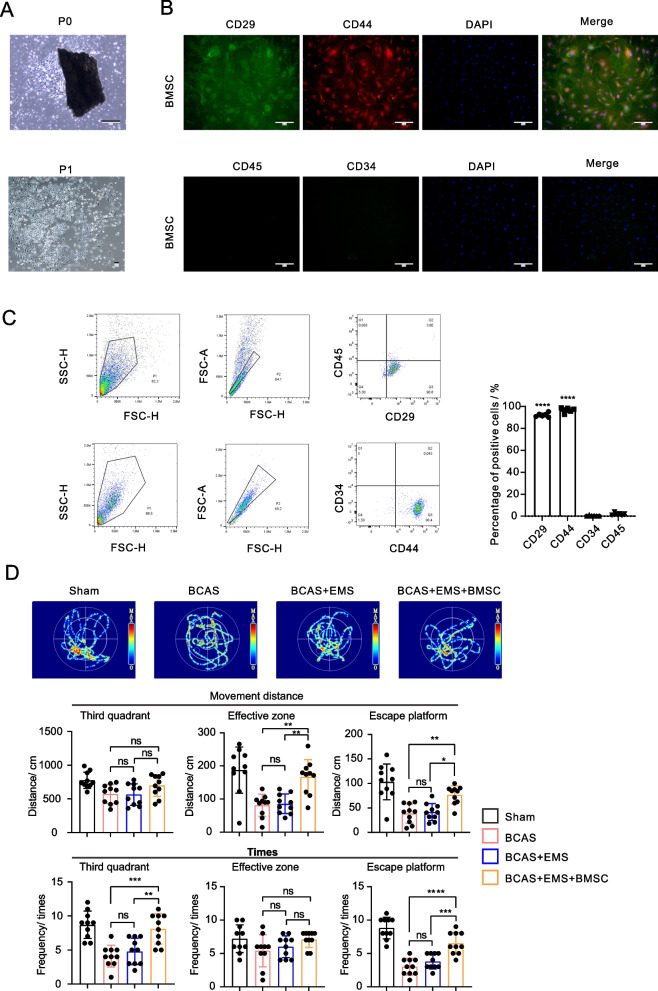
Local application of BMSCs improves cognitive function recovery in chronic brain ischemia treated with encephalo-myo-synangiosis. **A** Representative images of primary and subcultured mouse-derived BMSCs. Scale bar = 50 μm. **B** Representative images of immunocytochemical staining of mouse-derived BMSCs: CD29 (green), CD45 (green), CD34 (red) and CD44 (red). The merged image fuses red, green and blue fluorescent images. Scale bar = 50 μm. **C** Flow cytometry of mouse-derived BMSCs to detect CD29, CD45, CD34 and CD44. **D** The Morris water maze was used to measure the frequency and distance of mice entering the third quadrant, effective zone and escape platform 30 days after EMS and BMSC transplantation. (10 animals per group)

According to previously published protocols, primary bone marrow-derived MSCs (BMSCs) were extracted from murine bone chips and cultured as shown in Fig. 1A [20]. To verify the purification of the BMSCs, immunofluorescence staining of CD29, CD44, CD45 and CD34 was applied to the primary cultured cells (Fig. 1B). Moreover, flow cytometry was used to determine the BMSC-specific markers (CD29^+^ CD44^+^ CD45^−^ CD34^−^), and more than 90% of the cells belonged to this population (Fig. 1C), indicating the high purity of the BMSCs in the primary cultured cells.

Next, bilateral carotid artery stenosis (BCAS) was induced in mice to generate chronic brain ischemia. BMSCs were harvested, and cell depots were placed in the temporal muscle followed by EMS. Thirty days after BMSC transplantation, the cognitive behavior of the mice was assessed by the Morris water maze (MWM). Indeed, BCAS mice exhibited cognitive deficiency compared to the Sham group, and we observed a notable improvement in learning/memory capability in BMSC-transplanted littermates (Fig. 1D). Four groups were analyzed: the sham group, BCAS group, BCAS + EMS group, and BCAS + EMS + BMSC group.

### BMSC transplantation promotes encephalomyosinosynangiosis-mediated collateral neovascularization in BCAS mice

**Fig. 2:**
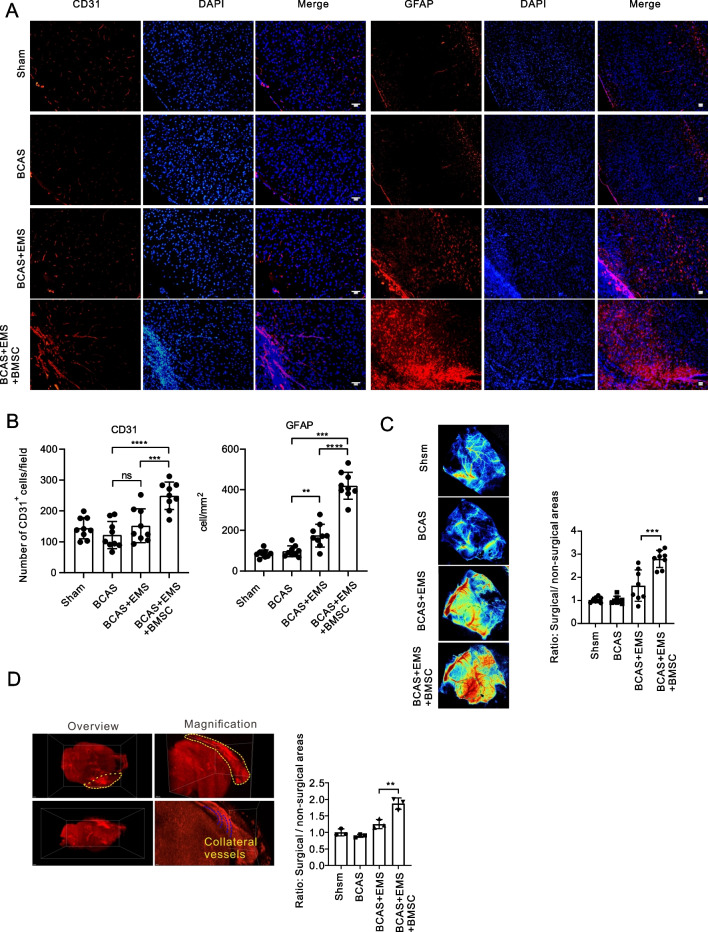
BMSC transplantation promotes encephalo-myo-synangiosis mediated collateral neovascularization in BCAS mice. **A**, **B** Representative images of immunofluorescence staining **A** and quantitative data of the number of blood vessels and astrocytes **B** in the contact between local temporal muscle and cerebral cortex 30 days after BMSC transplantation: CD31 (red), GFAP (red). The merged image fuses red and blue fluorescent images (9 animals per group). Scale bar = 50 μm. **C** Representative images of laser speckle imaging of regional cerebral blood flow and the relative blood flow of the surgical area compared with the nonsurgical area. (8 animals per group). **D** Representative images of 3D vascular structure in the attachment area of temporal muscle by light sheet microscopy

Since our hypothesis states that BMSC transplantation might enhance the angiogenesis capacity of EMS toward the brain, brain slices were then examined for collateral neovascularization on the EMS touching the brain surface. As expected, BMSC transplantation led to a significantly higher density of CD31-positive vessels (Fig. 2A, B). To further validate this observation, blood perfusion of the brain surface was examined via laser speckle imaging. Indeed, BMSC transplantation significantly amplified blood flow signals in BCAS mice (Fig. 2C). Furthermore, to clearly present the vascular structure of EMS-associated neovascularization after BMSC transplantation, brains were perfused with CD31 antibody and scanned by a light sheet microscope to elucidate the 3D vascular structure (Fig. 2D).

Interestingly, we examined astrocytes using immunofluorescence staining. To our surprise, BMSC transplantation induced significantly stronger astrogliosis between the brain and temporal muscle. (Fig. 2A).

Taken together, the above data demonstrated that BMSC transplantation largely improves EMS-mediated collateral neovascularization in BCAS mice.

### Local transplantation of BMSCs improves post-EMS neovascularization by secreting functional factors instead of differentiating into vascular cells

**Fig. 3:**
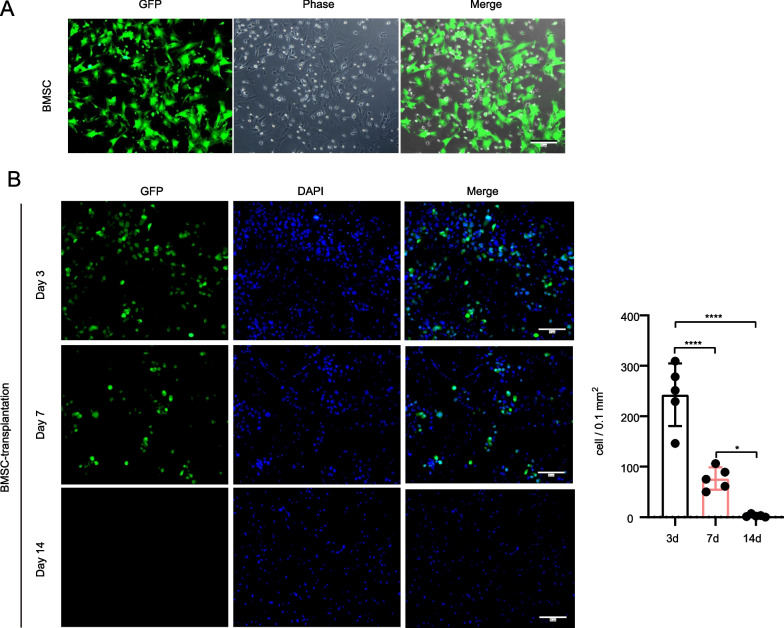
Tracing of local transplanted BMSCs. **A** Representative images of GFP reporter rats derived from BMSCs. The merged image fuses green fluorescent images and phase images. Scale bar = 50 μm. **B** Representative images of the number of GFP reporter rat-derived BMSCs surviving at different times in the temporal muscle attachment area. The merged image fuses green and blue fluorescent images. Scale bar = 50 μm

Previous reports have demonstrated the potential differentiation capacity of BMSCs into vascular cells, including endothelial cells, pericytes, or smooth muscle cells [25, 26]. To clarify whether the local transplantation of BMSCs exhibits a similar function, GFP fluorescence-labeled BMSCs were cultured from GFP reporter rats (Fig. 3A). Rat bilateral common carotid artery occlusion (BCCAO) was induced followed by EMS and BMSC transplantation. We found that the GFP signal remained up to 14 days after transplantation, which indicated that BMSCs could not survive more than 2 weeks after local transplantation and fail to differentiate into vascular cells under these circumstances (Fig. 3B). Therefore, we guessed that BMSCs affect angiogenesis post-EMS by secreting soluble cytokines.

**Fig. 4:**
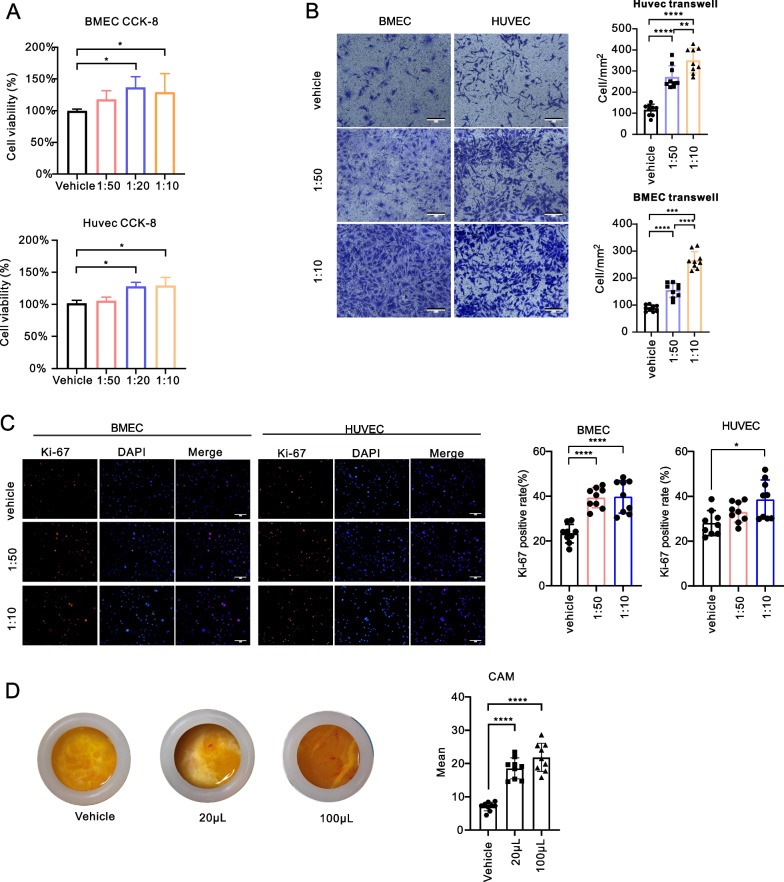
BMSC-CM promotes endothelial cell migration and proliferation. **A**–**C** CCK-8 (**A**), transwell (**B**) and ki-67 (**C**) results of BMECs and HUVECs stimulated by different concentrations of concentrated BMSC-CM. The merged image fuses red and blue fluorescent images. Scale bar = 50 μm. Chick chorioallantoic membrane assay results of BMECs and HUVECs stimulated by different concentrations of concentrated BMSC-CM. **D** Representative images and quantitative data of the chick chorioallantoic membrane assay after different doses of concentrated BMEC-sup were added to the chorioallantoic membrane

To validate this hypothesis, culture conditioned medium from BMSCs was collected and applied to brain microvascular endothelial cells (BMECs) and HUVECs. The results from CCK8 and Ki-67 staining showed significantly higher viability as well as proliferation ability of BMECs and HUVECs after concentrated BMSC-CM stimulation (Fig. 4A, C). In addition, increased migratory activity was also observed in endothelial cells treated with concentrated BMSC-CM (Fig. 4B). Furthermore, we evaluated the neovascularization of the chick embryo chorioallantoic membrane (CAM) after concentrated BMSC-CM treatment. As expected, an increasing number of vessels were found in the concentrated BMSC-CM treatment group (Fig. 4D).

**Fig. 5:**
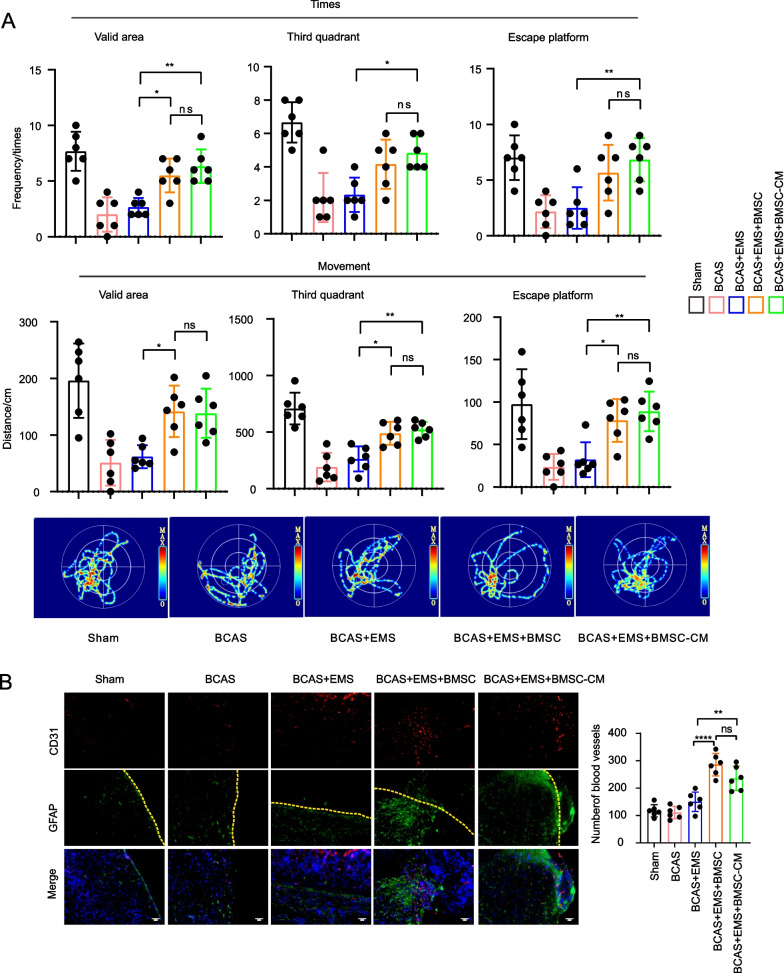
BMSCs and concentrated BMSC-CM can improve the behavior and angiogenesis of mice with chronic brain ischemia. **A** The Morris water maze was used to detect the frequency and distance of mice entering the third quadrant, effective zone and escape platform 30 days after EMS and concentrated BMSC-CM application. (6 animals per group). **B** Representative images of immunofluorescence staining and quantitative data of the number of blood vessels in contact between the local temporal muscle and cerebral cortex 30 days after concentrated BMSC-CM application: CD31 (red) and GFAP (green). The yellow dotted line represents the brain surface. The merged image fuses red, green and blue fluorescent images. (6 animals per group). Scale bar = 50 μm

Therefore, the supernatant of BMSCs was collected and concentrated. The concentrated BMSC-CM was then injected into the temporal muscle during EMS of BCAS mice. Motor function and cognitive behavior were assessed. Similar to BMSC transplantation, The BMSC-CM efficiently improved both motor function and cognitive behavior and increases the number of blood vessels at the surgical treatment area in BCAS mice (Fig. 5A, B). These data provide an improved method for future BMSC transplantation by using feasible supernatants to achieve robust treatment efficiency.

### Application of BMSCs and concentrated BMSC-CM increases the expression of pro-angiogenesis factors

**Fig. 6:**
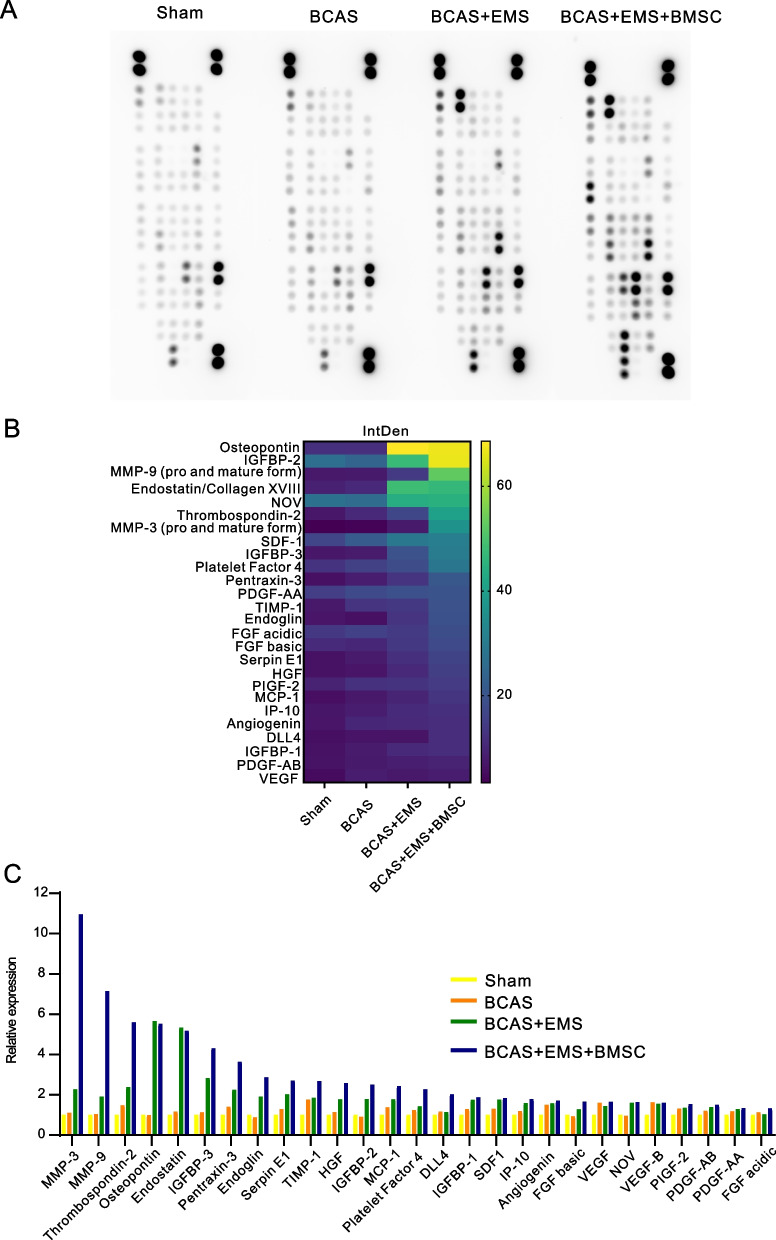
Angiogenesis array to screen potential pro-angiogenesis factors of MSC transplantation together with EMS surgery. **A** The dot plot shows proteome profiler (angiogenesis array) results in the cortex attached to the temporal muscle for each group. **B** The heatmap shows the intensity of the angiogenesis array. **C** The bar graph denotes the fold change of the dot intensity

To understand the potential mechanism of BMSC transplantation that facilitates neovascularization, we conducted the EMS surgery in combination with MSC transplantation on BCAS models. The cortex was collected for screening potential factors by Proteome profiler (angiogenesis array). As shown in Fig. 6A–C, several pro-angiogenesis factors (such as MMP-3, MMP-9, IGFBP-2 or IGFBP-3) were notably highly expressed in MSC transplantation group compared to others.

Moreover, we also examined the expression of pro-angiogenesis genes in the EMS part (including temporal muscle and its connecting brain cortex). Interestingly, the expression of known pro-angiogenesis genes, such as VEGF and TGF, was significantly elevated, and TGFα increased the most after concentrated BMSC-CM treatment (Additional file 1: Fig. S1A). At the same time, we also examined the expression of miRNA in the attached local tissues. In total, we found that there were differences in six miRNAs Additional file 1: Fig. S1B), among which miR-21a-5p, miR-877-5p, and miR-29a-3p were reported to be related to the function of mesenchymal stem cells in promoting angiogenesis [27–29], while miR-126a-3p and miR-221-3p were thought to be related to the function of endothelial cells [30, 31].

Taken together, BMSC transplantation or supernatant treatment upregulates the secretion of pro-angiogenesis factors.

## Discussion

Mesenchymal stem cell transplantation has been recognized as a promising therapeutic method for various diseases, including ischemic brain injury [32, 33]. Current strategies of MSC transplantation treatment against brain ischemia are mainly achieved by intravenous injection to repair the brain tissue damage caused by stroke [34–36]. Certain studies have also demonstrated intracranial or intrathecal delivery of MSCs [37, 38]. These studies suggest that MSC treatment alone results in significant treatment efficacy in animal models. Moreover, several clinical trials around the world have demonstrated the safety of MSC transplantation in brain ischemia [39]. In recent decades, EMS surgery has become popular and has resulted in superior curative effects in treating chronic brain ischemia, such as Moyamoya disease and middle cerebral artery stenosis [40, 41]. However, there is a lack of studies evaluating the effect of MSC transplantation in combination with surgery. Our present study provides evidence that local MSC transplantation remarkably improved post-EMS neovascularization.

Transplantation of stem cells can be done using either freshly prepared cells or cryopreserved cells, but cryopreservation can in some cases adversely affect adult stem cell (and stem cell-containing) populations, which may lead to therapeutic failure [42], therefore, freshly prepared stem cells were used in our study for the experiments.

The mechanisms by which MSCs promote neovascularization comprise multiple signals [43–45]. Due to their differentiation potential, MSCs are able to directly differentiate into cells participating in neovascularization, including pericytes, endothelial cells, and smooth muscle cells [46], which have been verified in the treatment of a variety of diseases. Numerous circumstances, such as hypoxia or inflammation, initiate MSC differentiation [47]. In our current study, MSCs were unlikely to differentiate into these vascular cells but were likely to secrete functional factors that promote angiogenesis. According to the our array result, multiple pro-angiogenesis such as MMP-3, MMP-9, IGFBP-2 or IGFBP-3 were expressed at much higher level in the MSC transplantation group. Indeed, Matrix metalloproteinases (MMPs) are proteases that exerts pro-angiogenesis effect by degradation of the vascular basement membrane, participating in extracellular matrix remodeling and releasing of angiogenic mediators [48]. Also, numerous studies revealed that Insulin-like growth factor binding proteins (IGFBPs), such as IGFBP-2, exert angiogenic function in both IGF and IGFR1 dependent pathways [49]. Our findings suggested the potential angiogenic mechanisms of MSC transplantation, while more detail investigations are needed to identify the major effectors. Moreover, previous studies revealed that MSCs release numerous proangiogenic growth factors, such as VEGF and PDGF [50]. In addition, MSC exosomal noncoding RNAs, such as miRNAs or circRNAs, were also shown to regulate endothelial cell gene expression, which facilitates vascular generation [51, 52]. Therefore, we also evaluated the gene expression of known pro-angiogenesis factors. Indeed, MSC or MSC supernatant transplantation notably increases VEGF, and TGF expression. Despite the well-known VEGF and TGFβ, our observation revealed TGFα as the most predominant upregulated factor after BMSC-CM treatment, indicating its crucial effect on neovascularization. Transforming growth factor alpha (TGFα) belongs to the TGF superfamily, and its structure is more similar to that of the EGF family [53, 54].There are no overwhelming investigations on the pro-angiogenesis impact of TGFα, while several studies have demonstrated its important role in stem cell self-renewal and differentiation.

Current strategies for MSC treatment are predominantly cell transplantation, which comes from the umbilical cord, bone marrow, or adipose tissue [55, 56]. Ethical problems or unpredicted tumorigenesis of MSC transplantation hinder the clinical application of MSC treatment [57]. Our study provides evidence that the supernatant of MSCs exhibits a pro-angiogenesis effect equivalent to that of cell transplantation in EMS, which further avoids the malpractice of cell transplantation. Furthermore, implementation of the supernatant application is easy to accomplish during EMS by injecting it onto temporal muscle [58]. Further studies are needed to examine whether transdermal injection to the temporalis muscle after EMS is applicable.

In summary, our present study revealed improvement of neovascularization and cognitive recovery in chronic brain ischemia post-EMS by using MSC as well as supernatant treatment, which provides a novel combination of therapeutic methods for the clinical treatment of chronic brain ischemia.

In addition, a limitation in the study was the randomization of the experimental animals into sham-operated and "model" groups during experimental grouping, which did not make sense because it automatically prevented allocation concealment. Moreover, the model used in the study only mimics vascular cognitive impairment to a certain extent, and we will use a combined model of vascular cognitive impairment for more in-depth exploration in future studies [59, 60].

## Conclusions

In conclusion, transplantation of MSCs during EMS can promote angiogenesis, and concentrated BMSC-CM can also achieve similar effects. Our study illustrated that MSC locally transplantation can be potential therapeutical options for improving EMS treatment efficiency which might be translated into clinical application.

### Supplementary Information

**Additional file 1: Fig. S1.** RT-qPCR results of angiogenic related mRNA (A) and miRNA (B) of cortex attached to the temporal muscle 30 days after EMS.

## Data Availability

The datasets used and/or analyzed in the current study are available from the corresponding author upon reasonable request.

## Abbreviations

MSC: Mesenchymal stem cells
BCAS: Bilateral carotid artery stenosis
EMS: Encephalo-myo-synangiosis
BCCAO: Bilateral common carotid artery occlusion
CCH: Chronic cerebral hypoperfusion
CCA: Common carotid arteries
