# Supplementary figures and images for: Local transplantation of mesenchymal stem cells improves encephalo-myo-synangiosis-mediated collateral neovascularization in chronic brain ischemia
Source: Stem Cell Res Ther. 2023 Sep 4;14:233. doi: 10.1186/s13287-023-03465-7 (PMC10478472; doi:10.1186/s13287-023-03465-7)

A

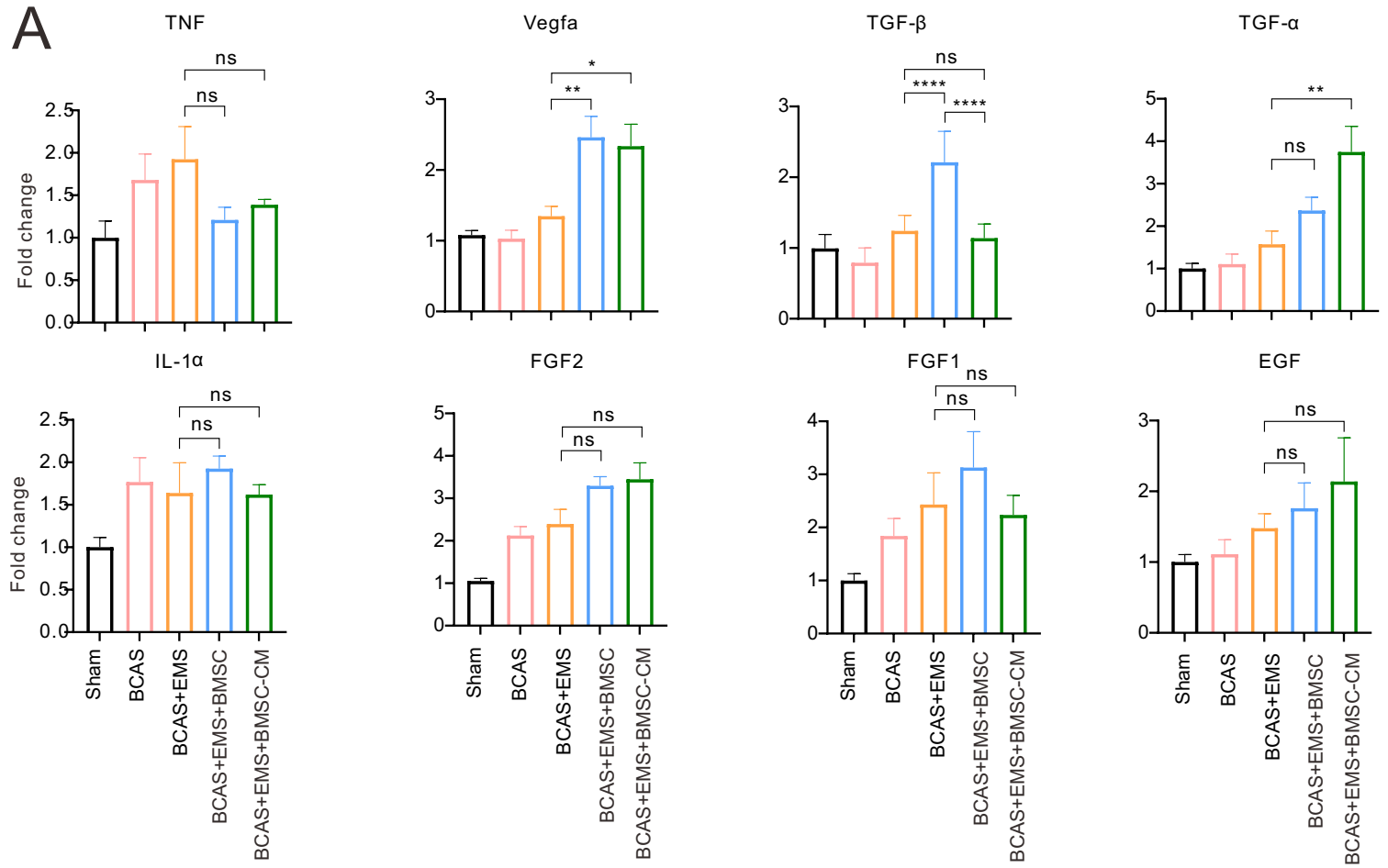

B

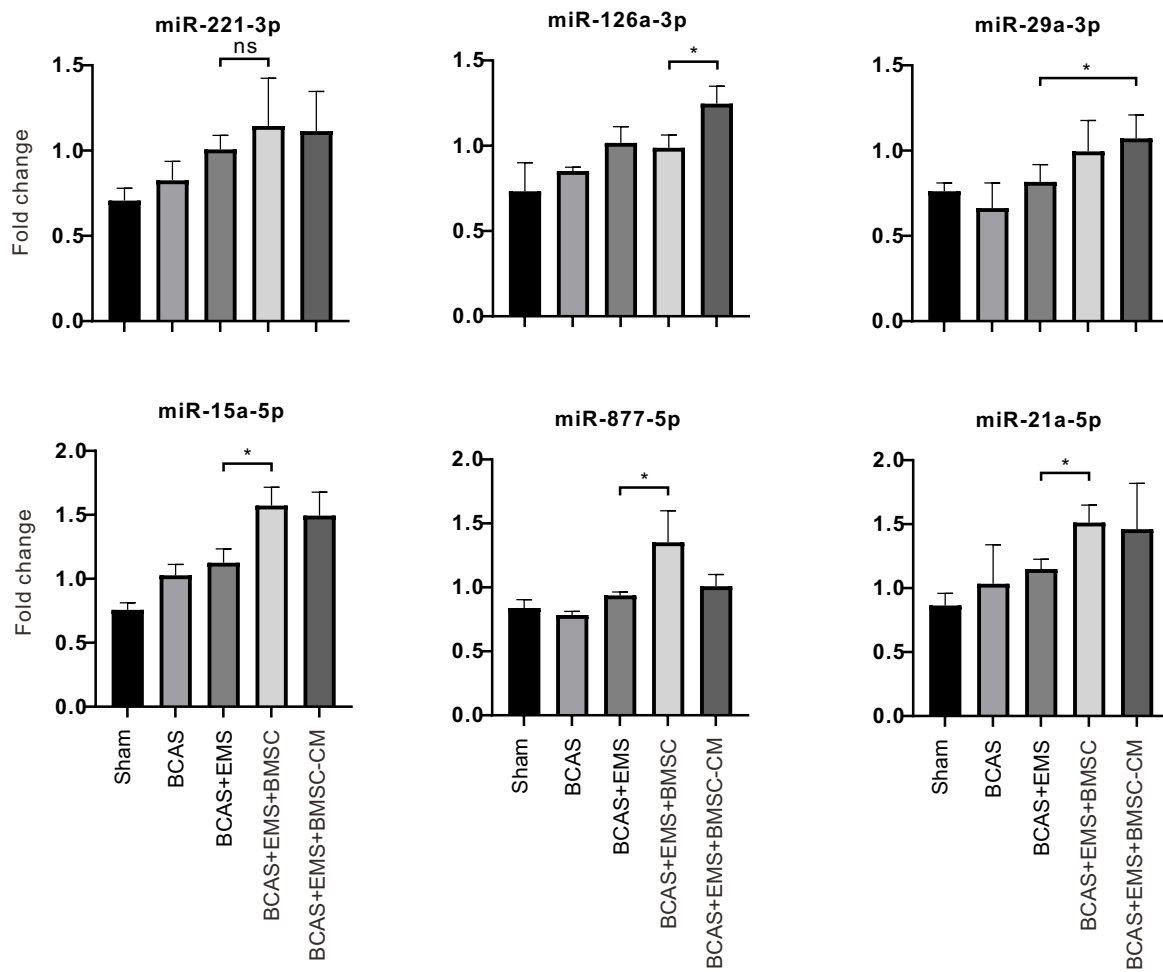

Supplement: Supplementary file 1 — Additional file 1: Fig. S1. RT-qPCR results of angiogenic related mRNA (A) and miRNA (B) of cortex attached to the temporal muscle 30 days after EMS. [file 13287_2023_3465_MOESM1_ESM.pdf]
